# The impact of clinically relevant health conditions on psychosocial outcomes in survivors of childhood cancer: results of the DCCSS-LATER study

**DOI:** 10.1007/s11764-024-01617-z

**Published:** 2024-06-22

**Authors:** Anne Maas, Heleen Maurice-Stam, Lieke E.A.M. Feijen, Jop C. Teepen, Alied M. van der Aa-van Delden, Nina Streefkerk, Eline van Dulmen-den Broeder, Wim J. E. Tissing, Jacqueline J. Loonen, Helena J. H. van der Pal, Andrica C. H. de Vries, Marry M. van den Heuvel-Eibrink, Cécile Ronckers, Sebastian Neggers, Dorine Bresters, Marloes Louwerens, Birgitta A. B. Versluys, Margriet van der Heiden-van der Loo, Leontien C. M. Kremer, Martha Grootenhuis, the Dutch LATER study group

**Affiliations:** 1 Princess Máxima Center for Pediatric Oncology, Utrecht, The Netherlands; 2 Amsterdam UMC/Location VUmc, Amsterdam, The Netherlands; 3 Beatrix Children’s Hospital, University of Groningen/University Medical Center Groningen, Groningen, The Netherlands; 4 Radboud University Medical Center, Nijmegen, The Netherlands; 5 Sophia Children’s Hospital, Erasmus Medical Center, Rotterdam, The Netherlands; 6 University Medical Center Utrecht, Wilhelmina Children’s Hospital, Utrecht, the Netherlands; 7 Division of Childhood Cancer Epidemiology, Institute of Medical Biostatistics Informatics and Epidemiology, University Medical Center of the Johannes Gutenberg University, Mainz, Germany; 8 Department of Medicine, Section Endocrinology, Erasmus Medical Center, Rotterdam, The Netherlands; 9 Willem Alexander Children’s Hospital/ Leiden University Medical Center, Leiden, The Netherlands; 10 Leiden University Medical Center, Leiden, The Netherlands; 11 Wilhelmina Children’s Hospital/University Medical Center Utrecht, Utrecht, The Netherlands; 12 Department of Pediatrics, Emma Children’s Hospital, Amsterdam UMC, University of Amsterdam, Amsterdam, The Netherlands

**Keywords:** Childhood cancer survivors, Cohort study, Health-related quality of life, Psychosocial functioning, Late effects, Health conditions

## Abstract

**Purpose:**

Investigate the association between presence, number and type of clinically relevant health conditions and a range of psychosocial outcomes (emotional, social, cognitive, physical) in survivors of childhood cancer (CCS).

**Methods:**

CCS from the Dutch Childhood Cancer Survivor Study (DCCSS)-LATER cohort (diagnosed between 1963–2001, attained age ≥ 18, diagnosed < 18, ≥ 5 years since diagnosis) completed a questionnaire on health conditions (2013–2014), and questionnaires on psychosocial outcomes (2017–2020): Hospital Anxiety and Depression Scale, Short form 36, TNO-AZL Questionnaire for Adult Health-Related Quality of Life, and the Self-Rating Scale for Post-Traumatic Stress Disorder. Associations among health conditions and psychosocial outcomes were assessed with regression analysis, adjusting for attained age, sex, and time since diagnosis, and adjusting for multiple testing (*p* < 0.004).

**Results:**

A total of 1437 CCS, mean age 36.3 years, 51.1% female, ≥ 15 years since diagnosis, completed questionnaires on health and psychosocial outcomes. CCS with a clinically relevant health condition, and those with more conditions had worse emotional, social, and physical outcomes; regression coefficients were small to moderate. CCS with gastro-intestinal conditions, endocrine, nervous systems, eye, or ear conditions, and especially those with secondary malignant neoplasms, reported worse psychosocial functioning; regression coefficients were small/moderate to large.

**Conclusion and implications:**

Health care professionals should be aware of the increased risk for psychosocial problems among CCS with health conditions, especially for survivors with secondary malignant neoplasms, gastro-intestinal, endocrine, nervous system, eye, and ear conditions. CCS may benefit from psychological interventions to develop coping strategies to manage health conditions and psychosocial consequences of the cancer trajectory.

**Supplementary Information:**

The online version contains supplementary material available at 10.1007/s11764-024-01617-z.

## Introduction

Survival rates for childhood cancer have significantly improved in recent decades, leading to an increasing population of survivors of childhood cancer (CCS) [1]. Although many years have gone by since their cancer experience, CCS remain at risk for long-term health consequences of their primary cancer and its treatments, including organ dysfunction and the development of second neoplasms [2–4]. A recent Dutch study found that nearly 50% of CCS had developed at least one clinically relevant health condition after a median of 18.5 years since diagnosis, and that CCS experienced 2.8 times more health conditions than siblings [5]. Clinically relevant health conditions are defined as morbidities with clinical symptoms and/or requiring medical treatment, and will be further referred to as health conditions [6]. Besides physical long-term health consequences, the cancer experience can have psychosocial consequences in CCS [7]. Although most CCS seem to function well psychosocially, subgroups experience lower quality of life or symptoms like anxiety, depression and post-traumatic stress [8–10].

Previous research has shown that cancer-related medical characteristics, such as type of diagnosis, age at diagnosis, and treatment, offer a limited explanation for variation in psychosocial outcomes, although it has been consistently observed that a CNS tumor diagnosis is associated with poorer outcomes [10–12]. The role of health conditions may be more prominent in explaining psychosocial functioning in CCS. Some cohort studies found that the presence of any late effect [13] and of any major medical condition [14] was associated with psychological distress, measured with the Brief Symptom Inventory. One cohort study suggests that health related quality of life (HRQOL) was most impaired in CCS with specific health conditions, such as memory problems, and musculoskeletal or neurological problems [12]. However, so far no studies investigated the association between specific health conditions and a wide range of psychosocial outcomes.

The present study aims to explore the association between clinically relevant health conditions (presence, number and specific type) and a wide range of psychosocial outcomes (emotional, social, cognitive, physical) in CCS.

## Methods

### Design and population

This study is part of the Dutch Childhood Cancer Survivor Study (DCCSS)-LATER study, which is a nationwide cohort study that included all CCS diagnosed between 1963 and 2001, aged < 18 years at diagnosis, ≥ 5 years since diagnosis at time of study, and treated in one of the seven former Dutch pediatric oncology centers [15].

The LATER study consists of two parts: the DCCSS-LATER 1 study (2013–2014) in which data on health conditions were collected [15], and the DCCSS-LATER 2 study (2017–2020) in which data on psychosocial outcomes were collected as part of the LATER Psycho-oncology sub-study [10, 16]. For the Psycho-oncology sub-study, CCS aged ≥ 18 years were eligible. The questionnaires on health conditions and psychosocial outcomes were completed from home, either digitally or using pencil and paper. Informed consent was obtained from all participants included in the study. The medical ethics board of all seven centers approved the study protocol.

## Measures

### Clinically relevant health conditions

A total of 75 clinically relevant health conditions were established, defined as health conditions that were symptomatic and/or for which medical intervention was required or recommended [5]. Health conditions were validated by self-reported medication use or medical record review. The validation process was conducted by comparing the information from the questionnaires with data on medication use and surgical treatment. If this information was not sufficient to determine whether the condition was present or not (e.g. if a condition was reported but no corresponding medication was listed), medical record data were obtained from the late effects outpatient clinics. Further details of the validation process, including a flowchart illustrating the steps, are described elsewhere [5]. The 75 clinically relevant conditions were clustered into 13 subtypes (Supplementary Table 1). We created a dichotomous variable indicating whether CCS had at least one clinically relevant health condition (presence health condition, yes/no), and a continuous variable indicating the number of clinically relevant health conditions.

### Psychosocial outcomes

In this study we explored the association between health conditions (measured in LATER 1) and various domains of psychosocial outcomes (measured in LATER 2) including psychological outcomes and HRQOL, specifically addressing emotional, social, cognitive, and physical domains. We operationalized these domains using the most relevant scales of various questionnaires (Table 1).

**Table 1:** Operationalization of emotional, social, cognitive, physical domains

Domain	Emotional	Social	Cognitive	Physical
*Instrument*	Scales			
*HADS*	Anxiety, Depression			
*SRS-PTSD*	Post-traumatic stress symptoms			
*TAAQOL*	Positive emotions	Social functioning	Cognitive functioning	Gross motor functioning, Sleep, Pain, Vitality, Daily activities
*SF-36*				General health perceptions

*HADS* Hospital Anxiety and Depression Scale, *SRS-PTSD* Self-Rating Scale for Post-Traumatic Stress Disorder, *TAAQOL: TNO-AZL* Questionnaire for Adult Health-Related Quality of Life, *SF-36* Short Form 36

#### Anxiety and Depression: HADS

The Hospital Anxiety and Depression Scale measures anxiety (7 items) and depression (7 items) in the past week (range 0–21) [17, 18]. Higher scores indicate more anxiety or depression. The Cronbach’s α for both subscales was good (0.81-0.83). The HADS has good psychometric properties [17].

#### Post-Traumatic Stress: SRS-PTSD

The Self-Rating Scale for Post-Traumatic Stress Disorder measures post-traumatic stress symptoms in the previous four weeks (0–17) [19]. Three clusters of symptoms were assessed: re-experiencing (5 items), avoidance (7 items) and hyper-arousal (5 items), and combined to a total score of post-traumatic stress symptoms (PTSS). The items of the questionnaire correspond to the diagnostic criteria for post-traumatic stress disorder (PTSD), as defined by the DSM-IV. When completing the SRS-PTSD, CCS were asked to take their childhood cancer in mind. The Cronbach's α of the total scale was good (α = 0.86). The SRS-PTSD has adequate psychometric properties [20, 21].

#### HRQOL: TAAQOL

The TNO-AZL Questionnaire for Adult Health-Related Quality of Life (TAAQOL) assesses HRQOL in several domains [22]. From the TAAQOL, we used eight scale scores (each 4 items): positive emotions, social and cognitive functioning, gross motor functioning, sleep, pain, daily activities, and vitality. The TAAQOL comprises two parts: the first part assesses the prevalence of health problems or limitations experienced in the past month, and the second part evaluates the emotional response to these problems or limitations. Respondents answered to both parts on a 4-point Likert scale, and each combination was given a single score between 0 and 4. The domain scores were then calculated and transformed to a 0–100 scale, with higher scores indicating better HRQOL. The internal consistency of the domain scores in the present study was acceptable to good, with Cronbach's alpha values ranging from 0.76-0.92. The TAAQOL has demonstrated adequate psychometric properties [22].

#### HRQOL: SF-36

The Dutch version of the Short Form 36 (SF-36) Health Survey assesses HRQOL over the past four weeks and consists of eight scales of which we have used the scale general health perceptions (5 items) [23]. The SF-36 scale scores were transformed to a score ranging from 0 to 100, with higher scores indicating better general health perceptions. The SF-36 shows good psychometric properties [24]. Cronbach’s α of the scale score was 0.85.

### Background characteristics

Sex was obtained via questionnaires in the DCCSS-LATER 2 study in the same period as the outcome measures were assessed. Attained age (birth month and year) and time since diagnosis were obtained from the DCCSS-LATER registry.

### Statistical analysis

Differences between participants and non-participants on socio-demographic and medical characteristics were tested using independent t-tests and Chi-Square tests, with Cohen’s d and Cramer’s V as effect sizes.

The association between health conditions and psychosocial outcomes was investigated with linear multiple regression analyses, adjusting for attained age, sex, and time since diagnosis.

Effect sizes of 0.2, 0.5. and 0.8 were considered small, moderate and large for mean differences between two groups (Cohen’s d) and for regression coefficients (beta’s) of dichotomous independent variables [25]. Effect sizes of 0.1, 0.3 and 0.5 were considered small, moderate and large for Cramer’s V and regression coefficients (beta’s) of continuous independent variables. Regression coefficients of small sizes, defined as ≤ 0.20 for dichotomous independent variables, and ≤ 0.10 for continuous independent variables, were not considered relevant and are therefore not discussed. To correct for multiple testing, a significance level of 0.004 was used for the regression analyses, corresponding to 0.05 divided by the number of 12 outcomes.

## Results

### Participants

**Fig. 1:**
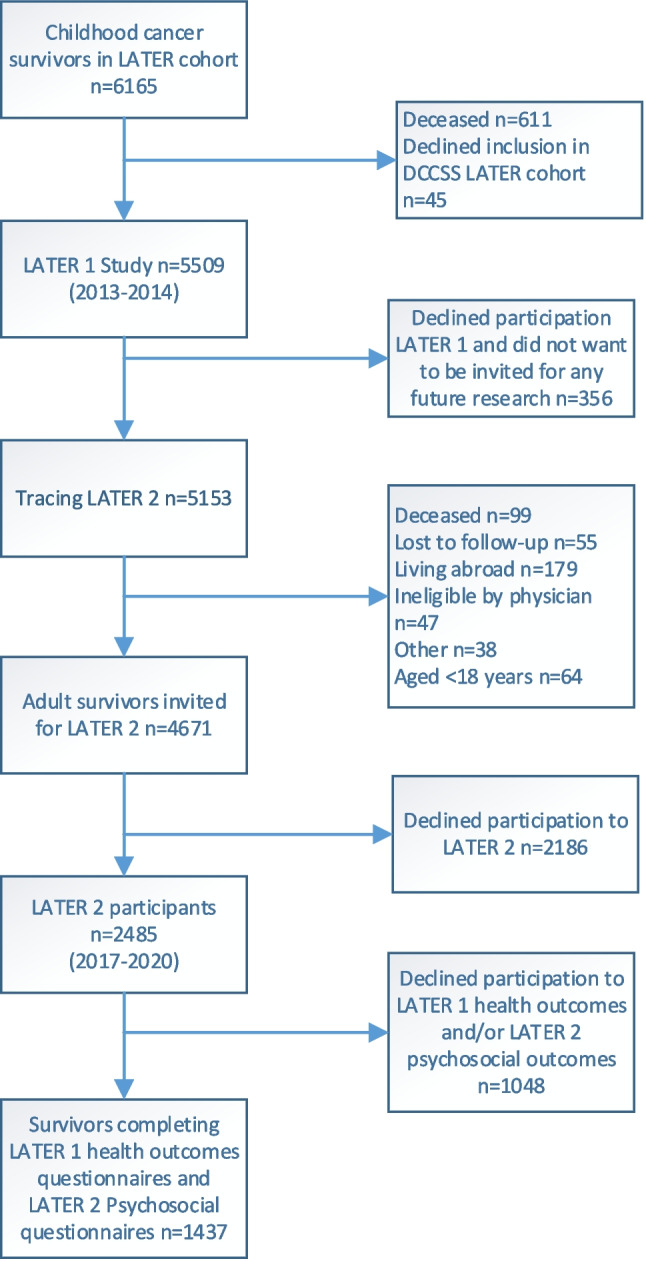
Flow of participants

The DCCSS LATER cohort comprised 6165 CCS of which 5455 were alive at time of invitation for the DCCSS-LATER 2 study (Fig. 1). A total of 4671 adult CCS were invited for the DCCSS-LATER 2 study of whom 2485 participated (53.2%). Of these 2485 CCS, 1437 (57.8%) CCS had completed questionnaires on health outcomes in LATER 1 as well as questionnaires on psychosocial outcomes in LATER 2.

Participating CCS had a mean age of 36.3 years (SD 9.6, range 18–71), 51% were female and mean time since diagnosis was 29.4 years (SD 8.6, range 15–55). Participants were compared to the 2514 (of the 3234) non-participants who did not decline the use of their data. Only small (V ≤ 0.12) differences were found between participants and non-participants on some socio-demographic and medical characteristics (Table 2).

**Table 2:** Demographic and Medical Characteristics CCS: Participants Versus Non-participants

	Participants (*N* = 1437)			Non-participants (*N* = 2514)			Cohen’s *d*
	*M*	SD	Range	*M*	SD	Range	
Age at study (years)	36.26	9.58	18.29–70.88	35.10	9.11	18.02–70.52	-0.12***
Age at first diagnosis (years)	6.84	4.75	0.00–17.96	6.59	4.70	0.00–17.95	-0.05
Time since first diagnosis (years)	29.42	8.56	15.34–55.01	28.51	8.22	15.44–56.18	-0.11**
	*% (N)*		*% (N)*		Cramer’s *V*		
**Socio-demographic characteristics**							
Sex					0.11***		
Male	48.9 (703)		60.2 (1513)				
Female	51.1 (734))		39.8 (1001)				
Partnered	79.5 (1010)						
Educational attainment							
Low	12.3 (174)						
Middle	41.4 (586)						
High	46.3 (655)						
Employed	84.6 (1199)						
**Medical characteristics**							
Clinically relevant health conditions (number)							
0	54.8 (787)						
1	27.2 (391)						
2	11.1 (159)						
3	4.6 (66)						
≥ 4	2.3 (34)						
Clinically relevant health condition (yes)	45.2 (650)						
Age at first diagnosis (years)							
0–5	52.7 (758)		53.9 (1355)		0.02		
6–11	27.6 (397)		28.5 (714)				
12–17	19.6 (282)		17.7 (445)				
Time since first diagnosis (years)					0.06*		
10–19	15.4 (222)		17.0 (428)				
20–29	40.7 (585)		43.0 (1082)				
30–39	31.0 (446)		29.2 (735)				
40–49	11.2 (161)		10.0 (251)				
50–59	1.6 (23)		0.7 (18)				
Recurrence (yes)	14.1 (202)		11.6 (291)		0.04*		
Diagnosis							
Hematologic	52.3 (713)		59.6 (198)		0.06*		
Central Nervous System	9.2 (126)		8.7 (29)		0.01		
Solid	38.5 (525)		31.6 (105)		0.06*		
Treatment							
Surgery (yes)	51.0 (732)		50.7 (1268)		0.00		
Chemotherapy (yes)	87.5 (1257)		82.2 (2064)		0.07***		
Radiotherapy (yes)	40.8 (586)		31.0 (778)		0.10***		

*Note.* *p-value < 0.05, **p-value < 0.01, ***p-value < 0.001, significant differences (*p* < 0.05) are presented in bold. Because of missing values, N varies slightly across variables. Data were missing for non-participating survivors who declined the use of their data in the DCCSS-LATER registry (N = 720)

### Associations between presence and number of health conditions and psychosocial outcomes

Table 3 and 4 present the regression models of the association between health conditions, defined as presence (yes/no) and number of conditions, and the psychosocial outcomes. The presence of health conditions and a higher number of conditions show a small association with more depressive symptoms and post-traumatic stress symptoms, worse social functioning, and lower quality of life on daily activities. It also shows a small to moderate association with worse gross motor functioning, vitality, and general health perceptions. The presence of a health condition is also associated with lower quality of life on pain. Supplementary Table 2 and 3 present the results by diagnosis (hematologic, CNS, and solid tumor). Associations between health conditions and psychosocial outcomes were small to moderate for each diagnosis group.

**Table 3:** Associations between presence & number of health conditions and psychosocial outcomes (domain emotional, social and cognitive), adjusted for age, sex and time since diagnosis

	Anxiety	Depression	PTSS ^a^	Positive emotions	Social functioning	Cognitive functioning
*N*	1366	1364	1204	1413	1413	1419
	*β (95% CI)*	*β (95% CI)*	*β (95% CI)*	*β (95% CI)*	*β (95% CI)*	*β (95% CI)*
Presence of health condition (dichotomous)	0.04 (-0.07; 0.15)	0.24*** (0.13; 0.35)	0.23*** (0.11; 0.34)	-0.16** (-0.27; -0.06)	-0.20*** (-0.30; -0.09)	-0.11* (-0.22; -0.01)
*R*^*2*^	0.02	0.02	0.02	0.01	0.02	0.03
Number of health conditions	0.02 (-0.03; 0.08)	0.13*** (0.08; 0.18)	0.11*** (0.05; 0.17)	-0.07* (-0.12; -0.01)	-0.12*** (-0.17; -0.07)	-0.06* (-0.11; -0.01)
*R*^*2*^	0.02	0.02	0.02	0.01	0.03	0.03

*P-value < 0.05, **p-value < 0.01, ***p-value < 0.001,

Regression coefficients of ≥ 0.20 for dichotomous independent variables, and ≥ 0.10 for continuous independent variables, with a *p*-value of < 0.004 are presented in **bold**

^a^*PTSS*  Post-traumatic stress symptoms

**Table 4:** Associations between presence & number of health conditions and psychosocial outcomes (domain physical), adjusted for age, sex and time since diagnosis

	Gross motor function	Daily activities	Sleep	Pain	Vitality	General health perceptions
*N*	1418	1414	1417	1414	1415	1381
	*β (95% CI)*	*β (95% CI)*	*β (95% CI)*	*β (95% CI)*	*β (95% CI)*	*β (95% CI)*
Presence of health condition (dichotomous)	-0.42*** (-0.53; -0.32)	-0.20*** (-0.31; -0.10)	-0.09 (-0.20; 0.01)	-0.21*** (-0.31; -0.11)	-0.34*** (-0.44; -0.24)	-0.49*** (-0.60; -0.39)
*R*^*2*^	0.13	0.04	0.05	0.10	0.08	0.09
Number of health conditions	-0.20*** (-0.25; -0.15)	-0.11*** (-0.16; -0.06)	-0.04 (-0.09; 0.01)	-0.09*** (-0.14; -0.04)	-0.19*** (-0.23; -0.14)	-0.28*** (-0.33; -0.23)
*R*^*2*^	0.13	0.04	0.05	0.09	0.09	0.11

^*^P-value < 0.05, **p-value < 0.01, ***p-value < 0.001,

Regression coefficients of ≥ 0.20 for dichotomous independent variables, and ≥ 0.10 for continuous independent variables, with a *p*-value of < 0.004 are presented in **bold**

### Associations between specific health conditions and psychosocial outcomes

Table 5 and 6 present the regression models examining the association between 13 specific types of health conditions and the psychosocial outcomes. Secondary malignant neoplasms, gastro-intestinal conditions, endocrine, nervous systems, eye, and ear conditions were significantly associated with at least two psychosocial outcomes (regression coefficients ≥ 0.2). Largest associations were seen between ear conditions and higher levels of depression (β 0.72), and worse social functioning (β -0.89). Large associations were also seen between musculoskeletal conditions and worse gross motor functioning (β -0.79), and between cardiac conditions and worse general health perceptions (β -0.80). Almost all conditions were associated with worse general health perceptions. Social functioning and physical domains (gross motor functioning, daily activities, vitality and general health perceptions) were associated with ≥ 3 specific types of health conditions.

**Table 5:** Associations between specific types of health conditions and psychosocial outcomes (domain emotional, social and cognitive), adjusted for age, sex and time since diagnosis

	Anxiety	Depression	PTSS ^b^	Positive emotions	Social functioning	Cognitive functioning
N	1377	1375	1215	1426	1426	1432
	*β (95% CI)*	*β (95% CI)*	*β (95% CI)*	*β (95% CI)*	*β (95% CI)*	*β (95% CI)*
**Specific type of health condition **^**a**^						
Secondary malignant neoplasms	0.48** (0.19; 0.77)	0.57*** (0.28; 0.86)	0.41* (0.10; 0.72)	-0.47** (0-.77; -0.18)	-0.52*** (-0.81; -0.23)	-0.28 (-0.57; 0.01)
Cardiac conditions	0.06 (-0.36; 0.24)	0.15 (-0.16; 0.45)	0.13 (-0.21; 0.46)	0.09 (-0.20; 0.39)	0.01 (-0.29; 0.30)	-0.13 (-0.42; 0.17)
Vascular conditions	-0.02 (-0.25; 0.22)	-0.04 (-0.28; 0.19)	-0.02 (-0.27; 0.24)	0.05 (-0.19; 0.28)	-0.09 (-0.33; 0.14)	0.19 (-0.04; 0.42)
Gastro-intestinal conditions	0.27* (0.01; 0.53)	0.35** (0.09; 0.61)	0.34* (0.05; 0.64)	-0.23 (-0.49; 0.03)	-0.27* (-0.53; -0.01)	-0.13 (-0.39; 0.12)
Respiratory conditions	-0.02 (-0.35; 0.31)	-0.25 (-0.58; 0.09)	-0.04 (-0.39; 0.32)	-0.04 (-0.37; 0.29)	0.24 (-0.09; 0.57)	-0.03 (-0.36; 0.30)
Renal and urinary tract conditions	0.06 (-0.27; 0.39)	0.21 (-0.12; 0.55)	0.31 (-0.04; 0.66)	-0.02 (-0.34; 0.31)	-0.27 (-0.60; 0.06)	-0.19 (-0.51; 0.13)
Hepatobiliary conditions	-0.40 (-0.84; 0.04)	-0.19 (-0.63; 0.25)	0.14 (-0.37; 0.66)	0.32 (-0.12; 0.76)	0.06 (-0.39; 0.51)	0.21 (-0.23; 0.65)
Musculoskeletal conditions	0.04 (-0.17; 0.25)	0.19 (-0.03; 0.40)	-0.00 (-0.23; 0.23)	-0.04 (-0.25; 0.18)	-0.06 (-0.27; 0.16)	0.02 (-0.19; 0.23)
Endocrine conditions (excluding obesity and underweight)	-0.02 (-0.16; 0.12)	0.15* (0.01; 0.29)	0.20** (0.05; 0.34)	-0.20** (-0.33; -0.066)	-0.19** (-0.32; -0.06)	-0.11 (-0.24; 0.03)
Nervous system Conditions	0.12 (-0.16; 0.40)	0.40** (0.12; 0.68)	0.40* (0.09; 0.70)	-0.14 (-0.42; 0.13)	-0.20 (-0.44; 0.08)	-0.32* (-0.59; -0.05)
Eye conditions	0.20 (-0.06; 0.46)	0.22 (-0.04; 0.48)	0.23 (-0.06; 0.52)	-0.01 (-0.26; 0.25)	-0.39** (-0.64; -0.13)	-0.22 (-0.47; 0.04)
Ear conditions	0.25 (-0.08; 0.57)	0.72*** (0.40; 1.05)	0.07 (-0.30; 0.43)	-0.37* (-0.70; -0.05)	-0.89*** (-1.21; -0.58)	-0.38* (-0.70; -0.06)
Other conditions	-0.22 (-0.76; 0.33)	-0.10 (-0.64; 0.45)	0.02 (-0.54; 0.57)	0.28 (-0.27; 0.82)	0.40 (-0.14; 0.95)	-0.25 (-0.77; 0.28)

^a^ All specific types of health conditions are analyzed in separate models ^b^ PTSS = Post-traumatic stress symptoms

*P-value <0.05, **p-value <0.01, ***p-value <0.001,

Regression coefficients of ≥ 0.20 for dichotomous independent variables, and ≥ 0.10 for continuous independent variables, with a *p*-value of < 0.004 are presented in **bold**

**Table 6:** Associations between specific types of health conditions and psychosocial outcomes (domain physical), adjusted for age, sex and time since diagnosis

	Gross motor function	Daily activities	Sleep	Pain	Vitality	General health perceptions
N	1431	1427	1430	1427	1428	1394
	*β (95% CI)*	*β (95% CI)*	*β (95% CI)*	*β (95% CI)*	*β (95% CI)*	*β (95% CI)*
**Specific type of health condition **^**a**^						
Secondary malignant neoplasms	-0.30* (-0.59; -0.02)	-0.41** (-0.70; -0.12)	-0.08 (-0.37; 0.21)	-0.03 (-0.31; 0.26)	-0.58*** (-0.86; -0.29)	-0.56*** (-0.85; -0.27)
Cardiac conditions	-0.25 (-0.53; 0.04)	0.17 (-0.13; 0.46)	-0.07 (-0.36; 0.22)	-0.00 (-0.29; 0.28)	-0.33* (-0.62; -0.05)	-0.80*** (-1.10; -0.51)
Vascular conditions	-0.02 (-0.25; 0.21)	-0.02 (-0.25; 0.22)	0.07 (-0.16; 0.29)	0.07 (-0.15; 0.30)	-0.10 (-0.33; 0.12)	-0.31* (-0.55; -0.07)
Gastro-intestinal conditions	-0.42** (-0.67; -0.17)	-0.18 (-0.43; 0.08)	-0.15 (-0.40; 0.10)	-0.44*** (-0.69; -0.19)	-0.48*** (-0.73; -0.23)	-0.51*** (-0.77; -0.25)
Respiratory conditions	-0.22 (-0.54; 0.10)	-0.00 (-0.33; 0.32)	-0.02 (-0.35; 0.31)	-0.16 (-0.47; 0.16)	-0.17 (-0.49; 0.16)	-0.46** (-0.80; -0.13)
Renal and urinary tract conditions	-0.39* (-0.71; -0.08)	-0.27 (-0.59; 0.06)	-0.14 (-0.46; 0.18)	-0.27 (-0.58; 0.04)	-0.16 (-0.47; 0.16)	-0.49** (-0.82; -0.16)
Hepatobiliary conditions	-0.24 (-0.67; 0.19)	-0.04 (-0.48; 0.40)	0.01 (-0.42; 0.44)	-0.12 (-0.54; 0.30)	-0.28 (-0.71; 0.15)	-0.53* (-0.97; -0.09)
Musculoskeletal conditions	-0.79*** (-0.99; -0.59)	-0.18 (-0.39; 0.03)	-0.21* (-0.42; -0.00)	-0.26* (-0.46; -0.06)	-0.26* (-0.47; -0.06)	-0.16 (-0.37; 0.06)
Endocrine conditions (excluding obesity and underweight)	-0.19** (-0.32; -0.06)	-0.23*** (-0.36; -0.09)	-0.05 (-0.18; 0.08)	-0.05 (-0.17; 0.08)	-0.25*** (-0.38; -0.12)	-0.40*** (-0.53; -0.26)
Nervous system conditions	-0.65*** (-0.92; -0.39)	-0.41** (-0.68; -0.14)	-0.20 (-0.47; 0.06)	-0.25 (-0.51; 0.02)	-0.45*** (-0.72; -0.19)	-0.67*** (-0.95; -0.39)
Eye conditions	-0.05 (-0.30; 0.20)	-0.17 (-0.43; 0.08)	0.00 (-0.25; 0.25)	-0.19 (-0.43; 0.06)	-0.15 (-0.39; 0.10)	-0.44*** (-0.70; -0.19)
Ear conditions	-0.16 (-0.47; 0.15)	-0.51** (-0.83; -0.19)	-0.16 (-0.47; 0.16)	-0.31 (-0.62; 0.00)	-0.41* (-0.72; -0.10)	-0.60*** (-0.92; -0.28)
Other conditions	-0.14 (-0.66; 0.37)	-0.14 (-0.69; 0.40)	-0.16 (-0.67; 0.36)	-0.56* (-0.108; -0.04)	-0.25 (-0.79; 0.28)	**-0**.36 (-0.90; 0.19)

^*^P-value < 0.05, **p-value < 0.01, ***p-value < 0.001,

Regression coefficients of ≥ 0.20 for dichotomous independent variables, and ≥ 0.10 for continuous independent variables, with a *p*-value of < 0.004 are presented in **bold**

^a^ All specific types of health conditions are analyzed in separate models

## Discussion

This study highlights the importance of considering the specific type of health condition when explaining psychosocial functioning in CCS. Specifically, we found that CCS with secondary malignant neoplasms, gastro-intestinal conditions, endocrine, nervous systems, eye, or ear conditions reported worse psychosocial functioning. Most impact of health conditions was seen on the social and physical domain.

Almost half of CCS experienced at least one health condition. A recent study in the DCCSS-LATER 1 cohort showed that CCS experienced 2.8 times more health conditions than siblings [5]. In this study we found only a small impact of the overall presence and number of health conditions on psychosocial outcomes. Other studies found an association between health conditions and worse psychosocial outcomes among CCS [12–14], but health conditions are operationalized and analyzed in various ways across studies which hinders a direct comparison of results. The fact that overall presence and number of health conditions only explained a small percentage of the variation in psychosocial functioning in CCS suggests that other factors play a more important role in explaining psychosocial functioning, such as how CCS cope with the cancer experience and its late effects [26–28], associated subjective appraisals [29, 30], and CCS’ received social support [31–33]. In future research, a biopsychosocial approach could be utilized [34], taking into consideration a range of physical, psychological and social factors when explaining psychosocial functioning in CCS.

The specific types of health conditions had a larger impact on psychosocial outcomes than the presence and number of any health condition. This indicates that the specific types of health conditions play a more prominent role in explaining psychosocial functioning in CCS. Psychosocial interventions tailored to different types of health conditions may be necessary to improve outcomes. Out of the specific types of health conditions, having had a second malignant neoplasm was associated with most psychosocial outcomes (6). We found that CCS with secondary malignant neoplasms experienced worse psychosocial outcomes, such as increased anxiety and depression, with a moderate effect size. These outcomes may be attributed to uncertainty regarding the prognosis or to the fear of recurrence (FCR) which has shown to be associated to anxiety and depression [35]. The results of a systematic review indicated that FCR is relatively stable over time and that interventions are therefore of importance [36]. CCS who have experienced a second neoplasm may benefit from (contemporary) cognitive behavioral therapies focusing on processes of cognition such as worry, rumination [37], and the disappointment arising from having to face cancer once again.

Furthermore, we found that having an ear condition, defined as deafness or hearing loss, had a large negative impact on social functioning, and a moderate to large impact on higher depression. It is important to realize that only severe hearing problems are taken into account and other ear conditions, such tinnitus, are not included. The results are in line with research in the general population showing that hearing problems are associated with more depression and loneliness in adults [38]. Since hearing problems are not exclusive to CCS, unlike FCR, CCS might benefit from interventions [39] available in the general population to address hearing problems.

Out of the psychosocial outcomes, the most impact of specific types of health conditions was observed on general health perceptions. Eight types of health conditions showed a moderate to large association with general health perceptions. Therefore, CCS’ perception of diminished health seems to reflect their actual health status.

Given the substantial percentage of CCS experiencing clinically relevant health conditions, and the potential impact of some of these health conditions on psychosocial functioning, international guidelines recommend CCS to attend lifelong follow-up care [40], which can play an important role in monitoring and addressing late effects of childhood cancer. Most late effects may not be preventable or reversible. CCS can therefore benefit from learning coping strategies in psychological interventions to manage physical and psychosocial consequences of the cancer trajectory, as early as possible [27]. Also, many CCS desire information about late effects such as what late effects to expect and how to deal with late effects, indicating the importance of adequate information provision, starting at diagnosis and continuing throughout survivorship care [41, 42].

### Strengths and limitations

This is the first cohort study on the presence, number and specific types of clinically relevant health conditions in relation to a wide range of psychosocial outcomes in long-term CCS. Major strengths are the unselected nationwide cohort, the detailed and reliable data regarding health conditions, and the comprehensive range of psychosocial outcomes covering emotional, social, cognitive and physical domains. This provided us with the opportunity to study the association between health conditions and psychosocial functioning in depth.

There are some considerations with the interpretation of the results. First, due to the time gap between the assessment of health conditions and psychosocial outcomes, changes may have occurred in CCS’ health status. While the vast majority of clinically relevant health conditions are permanent [5], CCS may have developed additional conditions in the meantime. Second, we have defined health conditions in various ways in our analysis (presence (yes/no), number, and specific types of health conditions). However, we did not assess the severity of health conditions, which might also affect psychosocial functioning in CCS, although we ensured the clinical relevance of all conditions. Additionally, health outcomes were self-reported and may not always be reported accurately. To mitigate this limitation, we validated the reported outcomes through self-reported medication use or by reviewing medical records. Third, even though this study had a large number of participants, some subgroups with specific types of health conditions were fairly small causing lower power to detect associations with psychosocial outcomes. Fourth, we have extensively examined the association between health outcomes and various psychosocial outcomes. Given the exploratory character of the study and the limited prior research on this topic, we opted for a moderately conservative correction for multiple testing to avoid overlooking potentially relevant associations between health conditions and psychosocial outcomes. Therefore, the possibility of false-positives should be taken into account when interpreting the results. Finally, this study included over half of the CCS invited for the LATER 2 study. We cannot rule out differences in health outcomes and psychosocial outcomes between participants and non-participants. However, we have not found important differences by diagnosis and treatment characteristics, and the presence of health conditions in these participants (45.2%, N = 1437) was in line with the presence in the total group that provided data on health conditions (46.4%, N = 3152) [5].

## Conclusion

Our study emphasizes the importance of considering the specific type of health condition when explaining psychosocial functioning in CCS. Specifically, we found that CCS with secondary malignant neoplasms, gastro-intestinal conditions, endocrine, nervous systems, eye, or ear conditions reported worse psychosocial functioning. This is a novel finding that points to the importance of psychosocial interventions tailored to different types of health conditions, which may be necessary to improve outcomes. CCS could benefit from psychological interventions to develop coping strategies to manage health conditions and psychosocial consequences of the cancer trajectory.

## Supplementary Information

Below is the link to the electronic supplementary material.

Supplementary file1 (DOCX 55.4 KB)

## Data Availability

The dataset analyzed during the current study is available from the corresponding author on reasonable request.
