# Supplementary material for: The impact of clinically relevant health conditions on psychosocial outcomes in survivors of childhood cancer: results of the DCCSS-LATER study
Source: J Cancer Surviv. 2024 Jun 22;19(6):2066–76. doi: 10.1007/s11764-024-01617-z (PMC12546303; doi:10.1007/s11764-024-01617-z)
Supplement: Supplementary file 1 — Supplementary file1 (DOCX 55.4 KB) [file 11764_2024_1617_MOESM1_ESM.docx]

| **Supplementary Table 1.** Overview Clinically Relevant Health conditions in CCS participants | | |  |
| --- | --- | --- | --- |
|  |  | % (N) |  |
| **Total** |  | 1437 |  |
| **Specific type of health condition** | **Outcome** |  |  |
| Secondary malignant neoplasms |  | 3.3 (47) |  |
| Cardiac conditions |  | 3.2 (46) |  |
|  | Ischemia | 0.3 (5) | |
|  | Heart failure | 2.0 (29) | |
|  | Pericarditis | 0.1 (2) | |
|  | Valvular disease | 0.4 (6) | |
|  | Arrhythmia | 0.6 (9) | |
| Vascular conditions |  | 5.7 (82) | |
|  | Thrombosis | 0.4 (6) | |
|  | Aneurysm | 0.1 (2) | |
|  | Hypertension | 5.0 (72) | |
|  | Other vascular conditions | 0.4 (6) | |
| Gastro-intestinal conditions |  | 4.5 (64) | |
|  | Gastroesophageal reflux disease | 4.0 (57) | |
|  | Inflammatory bowel disease | 0.3 (4) | |
|  | Other gastrointestinal conditions | 0.3 (4) | |
| Respiratory conditions |  | 2.5 (36) | |
|  | Obstructive pulmonary disease | 2.1 (30) | |
|  | Decreased pulmonary function | 0.3 (5) | |
|  | Other pulmonary conditions | 0.1 (1) | |
|  | Pulmonary resection/ transplantation | 0.1 (1) | |
| Renal and urinary tract conditions |  | 2.6 (37) | |
|  | Tubular dysfunction | 0.7 (10) | |
|  | Chronic kidney disease | 1.0 (14) | |
|  | Proteinuria | 0.1 (2) | |
|  | Nephrectomy | 0.1 (1) | |
|  | Renal transplantation | 0.2 (3) | |
|  | Urinary tract obstruction | 0.4 (6) | |
|  | Other conditions of kidney and urinary tract | 0.4 (6) | |
| Hepatobiliary conditions |  | 1.4 (20) | |
|  | Hepatitis C | 0.1 (1) | |
|  | Hemochromatosis | 0.1 (2) | |
|  | Liver transplantation | 0.1 (2) | |
|  | Cholecystectomy | 1.0 (15) | |
|  | Other Hepatobiliary conditions | 0.1 (1) | |
| Musculoskeletal conditions |  | 6.6 (95) | |
|  | Deformities | 1.5 (21) | |
|  | Amputation | 4.1 (59) | |
|  | Osteoporosis | 0.6 (9) | |
|  | Other musculoskeletal  Conditions | 1.0 (14) | |
| Endocrine conditions (excluding obesity and underweight) |  | 18.4 (263) | |
|  |  |  | |
|  | (Pan)hypopituitarism | 2.4 (35) | |
|  | Diabetes mellitus | 1.7 (25) | |
|  | Adrenal insufficiency | 1.1 (16) | |
|  | Hyperthyroidism | 0.3 (4) | |
|  | Hypothyroidism | 9.1 (130) | |
|  | Hyperparathyroidism | 0.1 (2) | |
|  | Hypoparathyroidism | 0.1 (2) | |
|  | Estrogen deficiency | 1.5 (21) | |
|  | Testosterone deficiency | 1.5 (22) | |
|  | Growth hormone deficiency | 3.1 (44) | |
|  | Diabetes insipidus | 0.8 (11) | |
|  | Prolactinoma | 0.1 (2) | |
|  | Polycystic ovarian syndrome | 0.1 (2) | |
|  | Pubertas tarda | 5.8 (83) | |
|  | Precocious puberty | 0.1 (2) | |
|  | Ovariectomy | 0.1 (2) | |
| Nervous system conditions |  | 3.7 (53) | |
|  | Epilepsy | 1.7 (25) | |
|  | Headache | 0.4 (6) | |
|  | Transient ischemic attack | 0.3 (4) | |
|  | Cerebrovascular accident- hemorrhagic | 0.1 (1) | |
|  | Cerebrovascular accident—ischemic | 0.3 (5) | |
|  | Hydrocephalus | 0.2 (3) | |
|  | Other neurological conditions | 1.2 (17) | |
| Eye conditions |  | 4.2 (61) | |
|  | Cataract | 3.7 (53) | |
|  | Eye removal | 0.6 (8) | |
| Ear conditions |  | 2.6 (38) | |
|  | Deafness | 0.1 (2) | |
|  | Hearing loss | 2.5 (36) | |
| Other conditions |  | 1.0 (14) | |
|  | Splenectomy | 0.2 (3) | |
|  | Hysterectomy | 0.6 (8) | |
|  | Mastectomy | 0.2 (3) | |

| **Supplementary Table 2.** Associations between presence & number of health conditions and psychosocial outcomes (domain emotional, social and cognitive), controlled for age, sex and time since diagnosis – Subgroup analysis diagnosis |
| --- |

*P-value <0.05, **p-value<0.01, ***p-value<0.001,

Regression coefficients of ≥ .20 for dichotomous independent variables, and ≥ .10 for continuous independent variables, with a p-value of < 0.004 are presented in **bold**

^a^ PTSS = Post-traumatic stress symptoms

|  |  | **Anxiety** | **Depression** | **PTSS** ^a^ | **Positive emotions** | **Social functioning** | **Cognitive functioning** |
| --- | --- | --- | --- | --- | --- | --- | --- |
|  |  | *β (95% CI)* | *β (95% CI)* | *β (95% CI)* | *β (95% CI)* | *β (95% CI)* | *β (95% CI)* |
| Hematologic | N | 704 | 704 | 627 | 711 | 709 | 712 |
|  | Presence of health condition (dichotomous) | .07  (-.09; .22) | .16*  (.01; .31) | .14  (-.02; .31) | -.12  (-.28; .03) | -.14  (-.30; .01) | -.08  (-.23; .07) |
|  | *R²* | .02** | .01 | .02** | .01* | .02*** | .03*** |
|  | Number of health conditions | .01  (-.06; .09) | .07  (-.01; .15) | .03  (-.06; .11) | -.04  (-.12; .04) | -.09*  (-.17; -.02) | -.04  (-.11; .04) |
|  | *R²* | .02** | .01 | .02** | .01* | .03*** | .03*** |
| Solid | N | 523 | 521 | 458 | 525 | 524 | 525 |
|  | Presence of health condition (dichotomous) | .03  (-.15; .21) | **.31*****  (.14; .49) | **.36*****  (.17; .55) | -.21*  (-.38; -.05) | -.19*  (-.35; -.03) | -.18*  (-.35; -.00) |
|  | *R²* | .01* | .02** | .05*** | .01 | .02** | .03*** |
|  | Number of health conditions | .05  (-.04; .13) | **.19*****  (.11; .27) | **.22*****  (.14; .31) | -.10*  (-.17; -.02) | **-.13*****  (-.20; -.06) | -.12**  (-.20; -.04) |
|  | *R²* | .02* | .04*** | .07*** | .01 | .04*** | .04 |
| CNS | N | 124 | 124 | 105 | 122 | 125 | 125 |
|  | Presence of health condition (dichotomous) | -.06  (-.41; .29) | .30  (-.08; .67) | .15  (-.25; .55) | -.11  (-.47; .26) | -.53*  (-.95; -.10) | -.05  (-.44; .33) |
|  | *R²* | .01 | -.01 | -.02 | -.02 | .05* | -.02 |
|  | Number of health conditions | -.01  (-.15; .14) | .13  (-.03; .29) | .09  (-.10; .28) | -.04  (-.20; .12) | -.23*  (-.40; -.05) | -.02  (-.18; .14) |
|  | *R²* | .01 | -.01 | -.02 | -.02 | .05* | -.015 |

|  | **Supplementary Table 3.** Associations between presence & number of health conditions and psychosocial outcomes (domain physical), controlled for age, sex and time since diagnosis – Subgroup analysis diagnosis | | | | | | |
| --- | --- | --- | --- | --- | --- | --- | --- |
|  |  | **Gross motor function** | **Daily activities** | **Sleep** | **Pain** | **Vitality** | **General health perceptions** |
|  |  | *β (95% CI)* | *β (95% CI)* | *β (95% CI)* | *β (95% CI)* | *β (95% CI)* | *β (95% CI)* |
| Hematologic | N | 712 | 710 | 711 | 711 | 711 | 707 |
|  | Presence of health condition (dichotomous) | **-.33*****  (-.46; -.21) | -.10  (-.25; .05) | .01  (-.14; .16) | -.20**  (-.34; -.06) | **-.29*****  (-.43; -.14) | **-.49*****  (-.63; -.34) |
|  | *R²* | .11*** | .03*** | .04*** | .07*** | .07*** | .10*** |
|  | Number of health conditions | **-.17*****  (-.24; -.11) | -.09*  (-.17; -.02) | .01  (-.07; .09) | -.07  (-.14; .01) | **-.17*****  (-.25; -.10) | **-.26*****  (-.33; -.19) |
|  | *R²* | .11*** | .04*** | .07*** | .07*** | .08*** | .11*** |
| Solid | N | 525 | 524 | 524 | 524 | 525 | 523 |
|  | Presence of health condition (dichotomous) | **-.49*****  (-.67; -.30) | **-.28****  (-.45; -.10) | -.25**  (-.42; -.08) | -.18*  (-.35; -.01) | **-.39*****  (-.56; -.22) | **-.47*****  (-.65; -.29) |
|  | *R²* | .15*** | .03*** | .06*** | .11*** | .08*** | .07*** |
|  | Number of health conditions | **-.22*****  (-.31; -.14) | **-.13****  (-.21; -.05) | -.11**  (-.19; -.04) | -.11**  (-.19; -.03) | **-.21*****  (-.29; -.14) | **-.29*****  (-.37; -.21) |
|  | *R²* | .15*** | .04*** | .06*** | .11*** | .09*** | .11*** |
| CNS | N | 124 | 125 | 125 | 124 | 124 | 122 |
|  | Presence of health condition (dichotomous) | -.37  (-.81; .06) | -.27  (-.66; .13) | .16  (-.21; .53) | -.39*  (-.75; -.03) | -.18  (-.54; .18) | -.46**  (-.81; -.12) |
|  | *R²* | .07* | .10** | .01 | .14*** | .01 | .04 |
|  | Number of health conditions | -.19*  (-.37; -.01) | -.07  (-.24; .10) | .05  (-.10; .21) | -.13  (-.29; .02) | -.08  (-.24; .07) | **-.26*****  (-.40; -.12) |
|  | *R²* | .08** | .09** | .01 | .12*** | .01 | .08** |

*P-value <0.05, **p-value<0.01, ***p-value<0.001,

Regression coefficients of ≥ .20 for dichotomous independent variables, and ≥ .10 for continuous independent variables, with a p-value of < 0.004 are presented in **bold**
